# Incidental finding of a hidden central polydactyly: A case report

**DOI:** 10.1002/ccr3.6867

**Published:** 2023-01-16

**Authors:** Mahir Bhoora, Dallan Dargan, Chris Milner

**Affiliations:** ^1^ University of York Hull York Medical School UK; ^2^ Hull University Teaching Hospitals NHS Trust, Department of Plastic Surgery Castle Hill Hospital Cottingham UK

**Keywords:** bifid distal phalanx, central polydactyly, congenital hand anomaly, hand trauma

## Abstract

Congenital hand anomalies are common, and must always be considered as a differential diagnosis in patients with hand pathology. We report the case of a child who sustained a fingertip injury to highlight an unusual presentation of central polydactyly.

## 1 INTRODUCTION

Polydactyly is a common congenital hand malformation, with an incidence of 0.3–3.6 in every 1000 live births, caused by defective patterning of the anterior–posterior axis of the developing limb bud.^1^ Polydactyly most frequently occurs as an isolated abnormality, however, rarely it may be present as part of a genetic syndrome.^2^ Non‐syndromic polydactyly may be classified as radial, ulnar, or central, depending on the location of the malformation^1^, ^2^; furthermore, polydactyly is a highly heterogenous condition, and deformities can range from a bifid distal phalanx to complete duplication of one or multiple fingers.^1^, ^3^

Central polydactyly is the congenital duplication of the index, middle, or ring fingers, and is much rarer than radial and ulnar polydactylies.^1^, ^2^ In the review by Wood^3^ of 144 patients with polydactyly, 22 (15%) had central polydactyly, and of these patients, 12 (55%) had a history of similar hand anomalies in family members, suggesting autosomal dominant inheritance. Twenty patients (91%) also had associated syndactyly. However, in the review of 12 patients with central polydactyly in Japan by Tada et al.^4^ no patients had a family history of similar anomalies.

We report the case of a child who sustained a fingertip injury to highlight an unusual presentation of central polydactyly, and the initial diagnostic uncertainty we faced in the context of the coincidental trauma.

## 2 CASE REPORT

**FIGURE 1:**
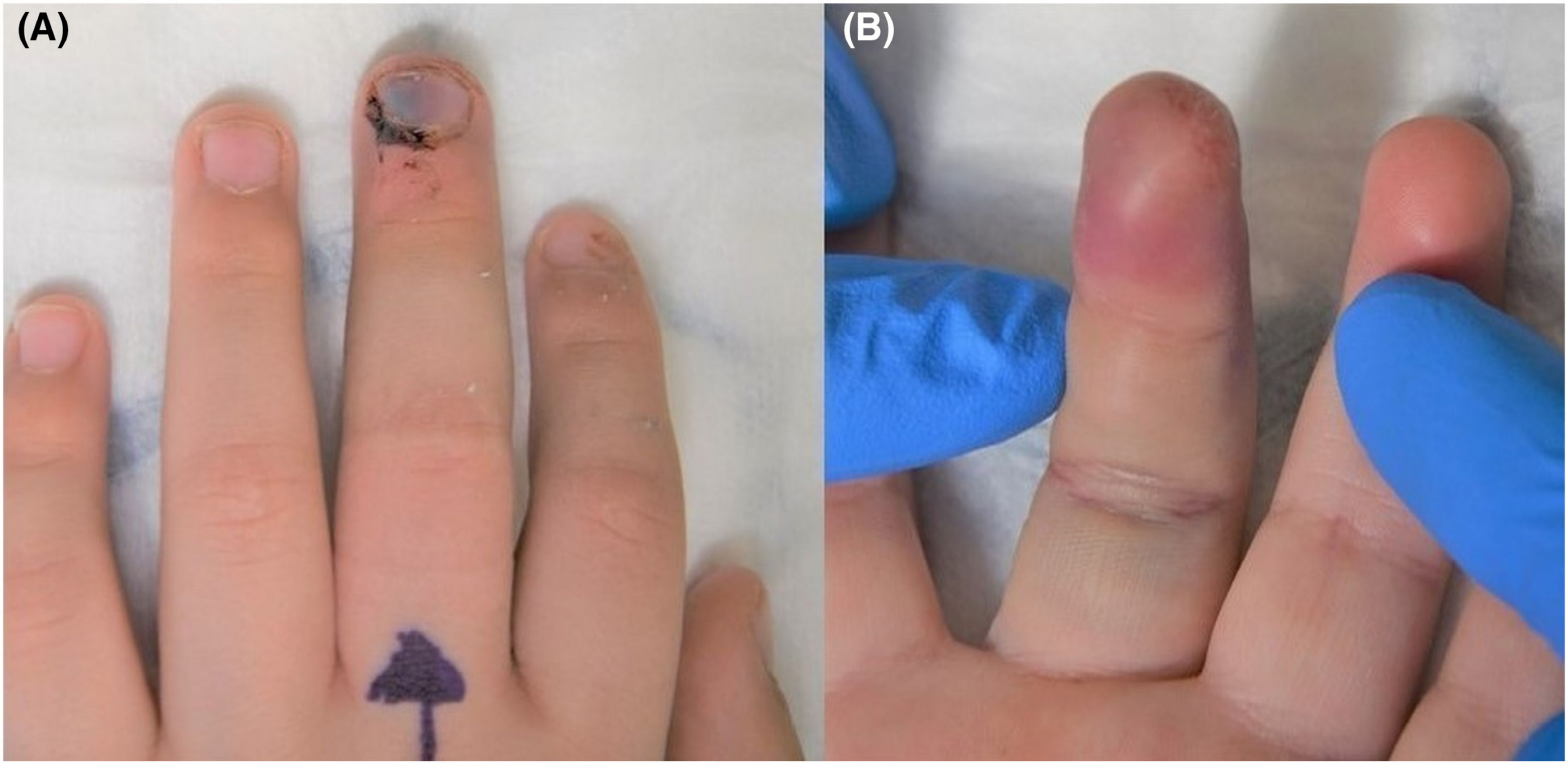
Preoperative clinical photographs of the dorsal (A) and palmar (B) aspects of the patient's left middle finger.

**FIGURE 2:**
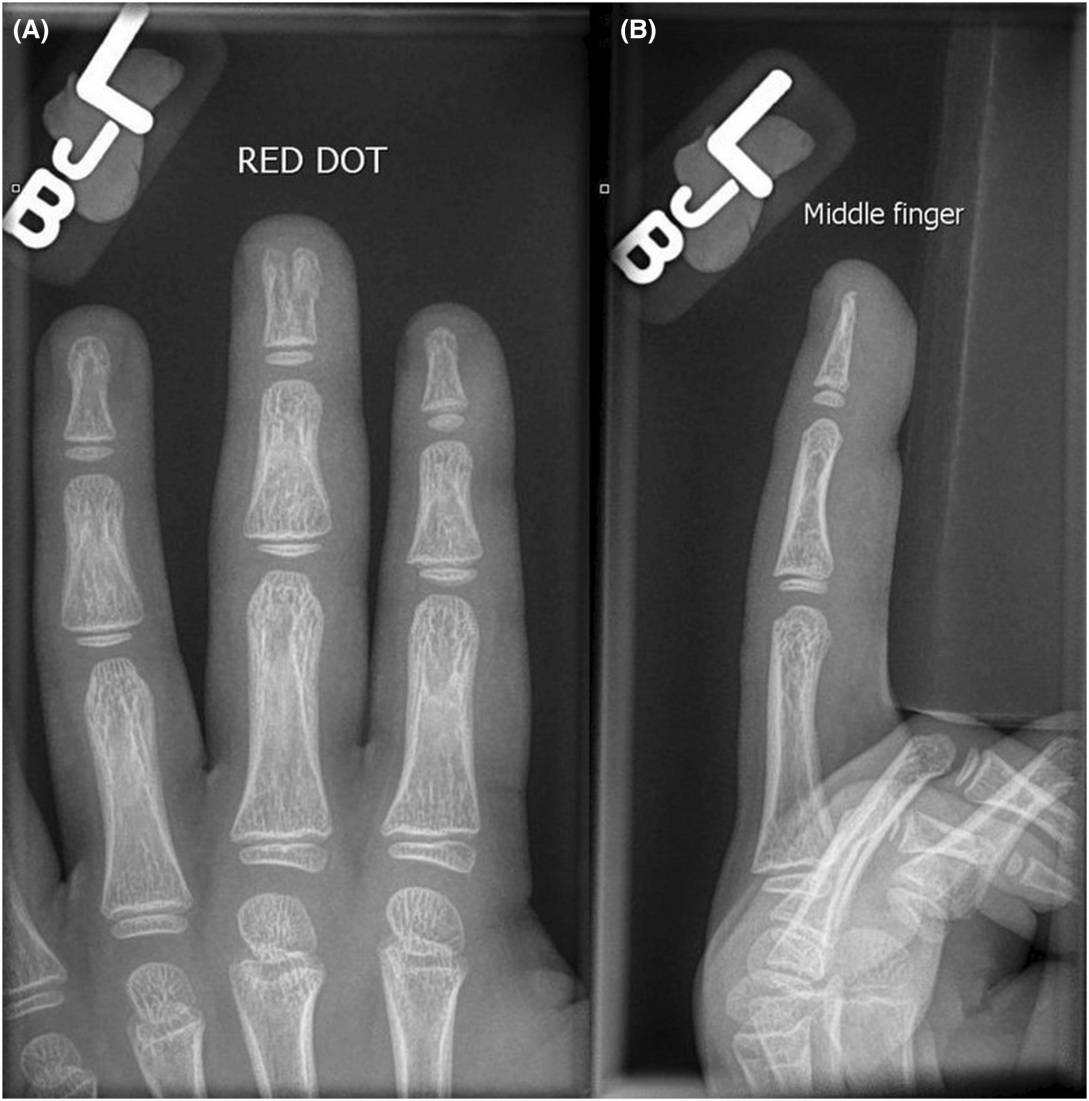
Anteroposterior (A) and lateral (B) view radiographs of the patient's left middle finger.

A 6‐year‐old Caucasian boy presented to our department, accompanied by his mother, 2 days after trapping his left middle finger in a car door. The child had not sustained any other injuries and was otherwise healthy. He had no significant past medical history or family history of any conditions. He was right‐hand dominant, lived with his mother and father at home, and attended primary school. Clinical examination of the left middle finger revealed an ulnar‐sided subungual hematoma, and an erythematous and tender fingertip (Figure 1A,B). Approximately 60% of the nail plate was elevated by the hematoma. There was no motor or sensory deficit, and no other fingers were injured. Radiographs of the patient's left hand demonstrated a central bone defect of the distal phalanx of the middle finger, and the waist of the distal phalanx was wider than those of the other digits. The bone either side of the central defect was well corticated, except for the fracture at the base of the radial ‘horn’ (Figure 2A,B). This case, therefore, presented a diagnostic dilemma as to whether the central bone defect was a fracture of the distal phalanx, or a congenital anomaly.

**FIGURE 3:**
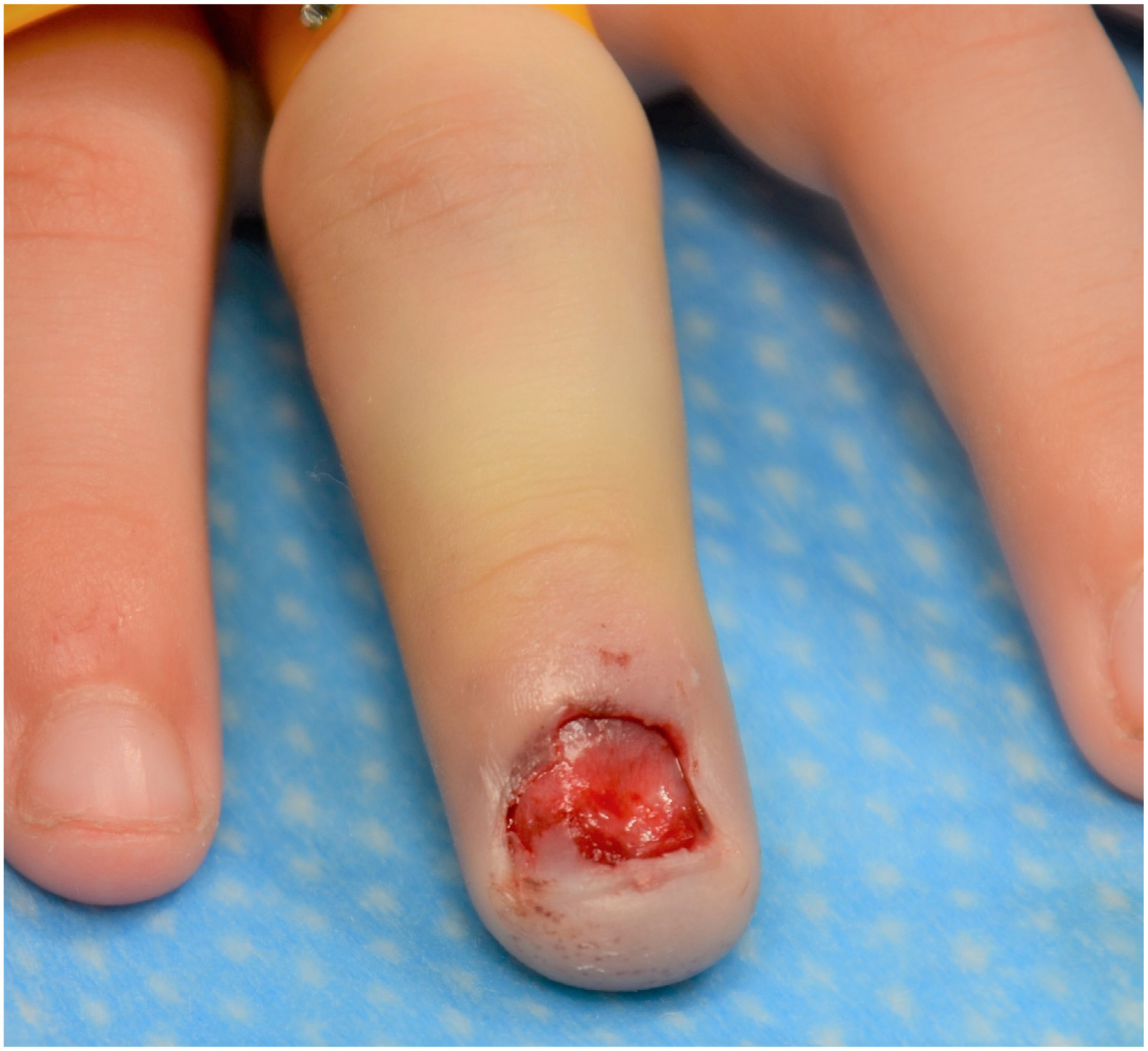
Clinical photograph of the patient's left middle finger after surgical removal of the nail plate.

The patient underwent a nailbed exploration under general anesthetic, and the operation was performed by a consultant plastic hand surgeon: the nail plate was removed, and while no nailbed lacerations were identified, there was a small central nailbed sulcus (Figure 3). Assessment of the distal phalanx of the left middle finger demonstrated solid underlying bone, with no crepitus or joint instability, and there were no observable or palpable abnormalities of any other fingers. On the basis of the clinical assessment and radiological investigations, a diagnosis of congenital bifid distal phalanx of the left middle finger was made. After recovering from the operation, the patient was discharged home the same day, with no formal post‐operative follow‐up required.

## 3 DISCUSSION

Considering the radiological appearance of our patient's left middle finger and the observable nailbed sulcus between the two bone ‘horns’ of the bifid distal phalanx, we believe to have identified a subtle, asymptomatic central polydactyly, brought to our attention only by the coincidental traumatic injury. As far as the authors are aware, this is a unique presentation for the diagnosis of this extremely rare congenital anomaly.

**FIGURE 4:**
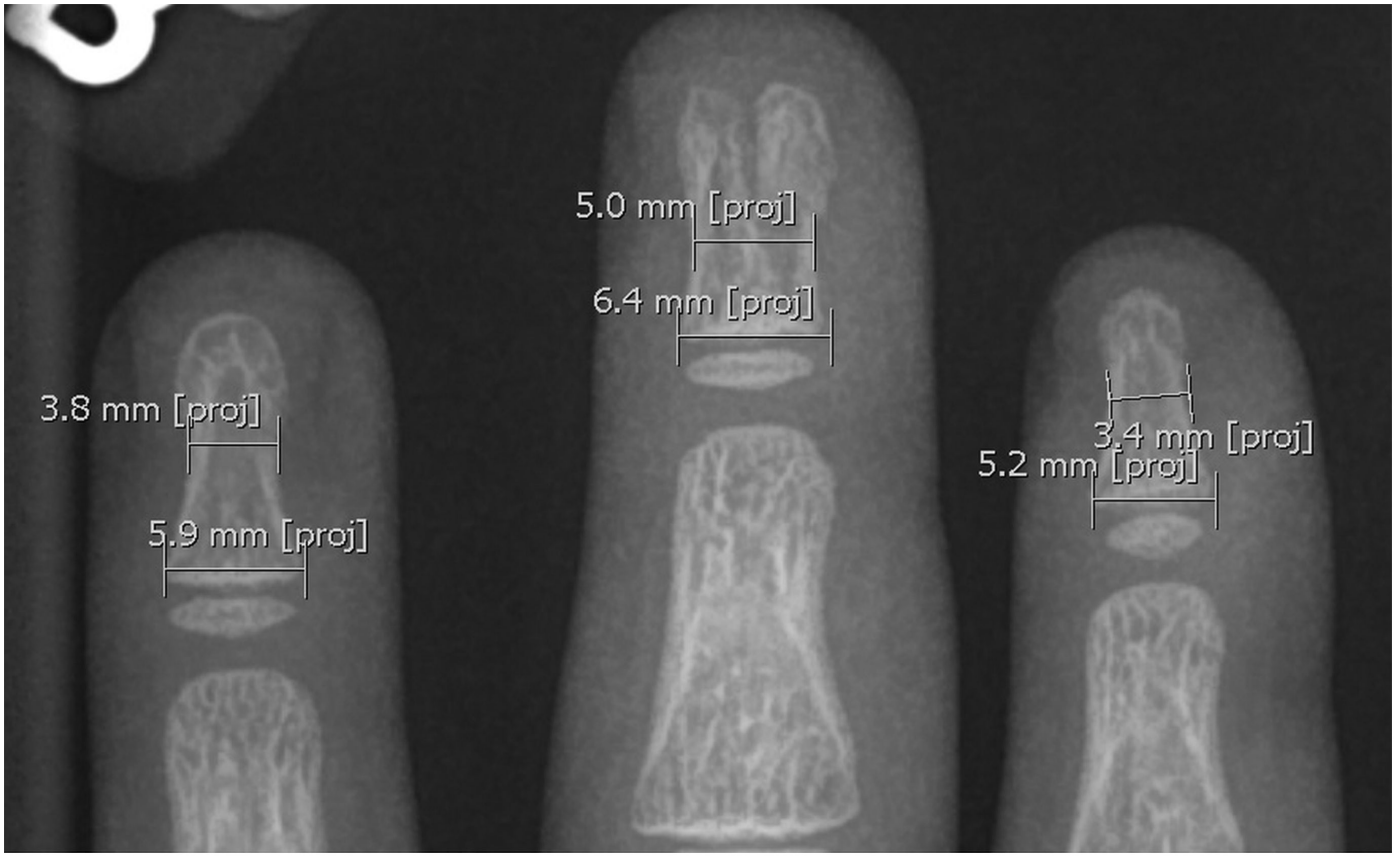
Measurements (in millimeters) of the waist widths and base widths of the patient's distal phalanges of the left index, middle, and ring fingers.

While tuft fractures are common injuries following trauma to the fingertips,^5^ for the apparent coronal plane separation of the distal phalanx to have been caused by a fracture, it would have been necessary for an enormous and precise shearing force to have been applied across the fingertip, however, this would have also resulted in significant soft tissue damage. Furthermore, measurements of the waist widths and base widths of the patient's distal phalanges of the left index, middle, and ring fingers (Figure 4) reveal that the distal phalanx of the middle finger is proportionally wider than the distal phalanges of the other two fingers (Table 1). The absence of skin or nailbed lacerations in our patient, plus the remarkably wide distal phalanx of the middle finger, therefore demonstrate that the bony abnormality identified radiologically predates the presenting trauma. While radiological investigation of the child's right hand and feet would have provided a greater insight into their polydactyly, clinically, these were not indicated. Additionally, a face‐to‐face post‐operative follow‐up appointment to closely examine the patient's hands and feet was not seen as essential in the context of the COVID‐19 pandemic.

**TABLE 1:** Measurements and ratios of the base widths and waist widths of the patient's distal phalanges

Left‐hand distal phalanx	Waist width (mm)	Base width (mm)	Waist width: base width ratio
Index finger	3.4	5.2	1: 1.529
Middle finger	5.0	6.4	1: 1.280
Ring finger	3.8	5.9	1: 1.553

Abbreviation: mm, millimeters.

Surgery for central polydactyly has been regarded as difficult, especially when complicated by co‐existing syndactyly, and patients require individualized treatment to create a functional and aesthetic hand.^2^, ^3^ Fortunately, in our case, the patient's degree of central polydactyly was extremely mild, asymptomatic, and cosmetically invisible, hence there was no indication for further investigation or surgical management. Moreover, the absence of syndromic features, co‐existing congenital abnormalities, or a family history of congenital hand anomalies further suggest that this child's central polydactyly occurred as a rare, isolated abnormality.

## 4 CONCLUSION

Injuries to the hand are common, but it is critical for clinicians to assess all patients individually to ensure accurate diagnosis. We report this case to highlight the unusual presentation of a rare congenital hand anomaly masquerading as a common traumatic injury, and the initial diagnostic uncertainty we faced. Methodical clinical examination combined with careful interpretation of the radiological images were essential in determining the correct diagnosis, and therefore planning appropriate management. We recommend that clinicians consider the possibility of a congenital bifid distal phalanx in patients who have sustained fingertip trauma and there appears to be a longitudinal split fracture, but no soft tissue damage is present.

## AUTHOR CONTRIBUTIONS

**Mahir Bhoora:** Conceptualization; data curation; formal analysis; investigation; methodology; project administration; visualization; writing – original draft; writing – review and editing. **Dallan Dargan:** Conceptualization; investigation; writing – review and editing. **Chris Milner:** Conceptualization; investigation; supervision; validation; writing – review and editing.

## FUNDING INFORMATION

The authors received no financial support for the research, authorship, and/or publication of this article.

## CONFLICT OF INTEREST

The authors declare no potential conflicts of interest with respect to the research, authorship, and/or publication of this article.

## ETHICAL APPROVAL FOR STUDY

Given the case report nature of this publication, ethical approval from a local ethics committee was not required. All procedures followed were in accordance with the ethical standards of the responsible committee on human experimentation (institutional and national) and with the Declaration of Helsinki 1975, as revised in 2008.

## CONSENT

Written informed consent was obtained from the patient's parent.

## Data Availability

Data sharing not applicable to this article as no datasets were generated or analysed during the current study.

## References

[1] Umair M , Ahmad F , Bilal M , Ahmad W , Alfadhel M . Clinical genetics of polydactyly: an updated review. Front Genet. 2018;9(447):1‐9. 30459804 10.3389/fgene.2018.00447 PMC6232527

[2] Comer GC , Potter M , Ladd AL . Polydactyly of the hand. J Am Acad Orthop Surg. 2018;26(3):75‐82. 29309292 10.5435/JAAOS-D-16-00139

[3] Wood VE . Treatment of central polydactyly. Clin Orthop Relat Res. 1971;74:196‐205. 4322173

[4] Tada K , Kurisaki E , Yonenobu K , Tsuyuguchi Y , Kawai H . Central polydactyly—a review of 12 cases and their surgical treatment. J Hand Surg Am. 1982;7(5):460‐465. 7130654 10.1016/s0363-5023(82)80040-3

[5] Oetgen ME , Dodds SD . Non‐operative treatment of common finger injuries. Curr Rev Musculoskelet Med. 2008;1(2):97‐102. 19468880 10.1007/s12178-007-9014-z PMC2684218

